# The impact of biofertilizer technology adoption on the faba bean productivity in the central highlands of Ethiopia: The propensity score matching model

**DOI:** 10.1371/journal.pone.0333105

**Published:** 2025-09-29

**Authors:** Getachew Chala, Bezabih Emana, Daniel Masresha

**Affiliations:** 1 Economics Department, Business and Economics College, University of Wollega, Nekempte, Ethiopia; 2 General Managers HEDBEZ Business & Consultancy PLC, Addis Ababa, and Haramaya University, Dire Dawa, Ethiopia; Graphic Era Institute of Technology: Graphic Era Deemed to be University, INDIA

## Abstract

Adoption of organic fertilizer technology increases crop productivity through replenishing the soil with natural organic nutrient as compared to in-organic chemical that is costly and damaging the soil fertility in the long run. Few studies have been documented on the contribution of bio-fertilizer on crop productivity on the plots of smallholder households. This study investigates the impact of adoption of bio-fertilizer technology on the faba bean productivity in the central highlands of Ethiopia, particularly in rural districts surrounding Addis Ababa, Ethiopia. The study is limited to small holder faba bean farmers in the central highlands of Ethiopia due to the diversified agro-ecology, socioeconomic, cultural and institutional factors in the country; it would be difficult to conduct a study on a specific area and generalize for others. A multi-stage sampling method was utilized to select 384 households for the sample. The primary data for the study was collected with a face to face interview using structured questionnaire. Propensity score matching (PSM) technique was employed to evaluate the impact of adoption of bio-fertilizer on faba beans productivity in the study area. The finding of the research revealed that, biofertilizer adoption on average increased faba bean productivity of adopters by about 5.1 quintals ha^-1^ than the non-adopters. Thus, based on the finding of the study, it is recommendable to strengthen existing extension service to promote the use of biofertilizer.

## 1. Introduction

About 80% of Ethiopians work in agriculture, which is the country’s main source of income despite the country’s far from self-sufficient food production. But Ethiopian smallholder farmers have very little economic resources, so neither mechanized farming nor fertilizer purchases are feasible. Because of this, it is imperative to employ intensive rehabilitation and sustainable management techniques that can support higher crop productivity (yields) and soil protection. Few Ethiopian farmers have adopted modern technology in farming systems to date, despite research pointing to numerous prospective pathways for its introduction [1].

Ethiopia, a significant economic contributor to food production, has been urged to raise agricultural output together with other African farmers. However, among the main causes, crop yield has been severely impacted by unfavorable economic conditions, a lack of technological advancement, and inefficient farming methods [2]. A substantial amount of nutrients are being lost from African soils, especially Ethiopia, as a result of the numerous ecological harms brought on by the over use of chemical fertilizers. These effects are typically irreversible and uncontrollable [3]. Additionally, there has been a proposal to modify the application of agrochemical inputs or at the very least, to decrease their usage, driven by the increasing demand for high-quality food products and sustainable agricultural practices. Therefore, the discovery and application of biofertilizers as an alternative has resulted from the search for a cost-effective, environmentally friendly, and sustainable means of enhancing plant growth and output [4]. Biofertilizers, which consist of beneficial live bacteria combined with cost-effective carrier materials, enhance the growth and development of plants when introduced to the soil and seeds. These biologically active formulations work by augmenting the availability of essential nutrients that promote plant growth [5,6].

Therefore, biofertilizer has a great economic importance in improving the productivity of smallholder farmers in Ethiopia [7].Indicators of revenue and productivity have frequently been utilized as the primary motivators for farmers to adopt new technology [8]. Thus the objective of this study is to analyze the impact of biofertilizer technology adoption on the faba bean productivity of smallholder farm households in rural districts surrounding Addis Ababa, which is found in the central highlands of Ethiopia. The study employed cross-sectional data obtained through household interviews in the study area based on the information generated from the sample households’ survey during a single cropping season. The study is limited to the small holder faba bean farmers in the central highlands of Ethiopia mainly due to the diversified agro-ecology, socioeconomic, cultural and institutional factors in the country; it would be difficult to conduct a study on a specific area and generalize for others.

The findings of the research will be of great policy use in the current agricultural technology development plan for attaining food-self sufficiency and poverty reduction in the country and the study area being one of the strategic implementation areas. The result is important in providing information that will enable the agricultural research centers continue providing biofertilizer technology and modifying the approaches of its extension. The outcome of the study also helps the policy makers in formulating appropriate biofertilizer technology adoption policies for the agricultural sector. One specific policy measure could be to increase the number and caliber of extension workers or development agents who regularly and directly interact with smallholder farm households through training and other means. Most importantly, it can serve as a standard for future research on the factor affecting the farmer’s decision to adopt the technology.

## 2. Research methodology

### 2.1. Sampling and data collection techniques

This study was held in the central highlands of Ethiopia, Particularly in rural districts surrounding Addis Ababa; the capital city of the country. The study area is located between 8°25°N and 9°25°N in latitude and between 38°25°E and 39°07°E in longitude. A three-stage sampling methodology was employed to choose a representative sample. First, two major faba bean growing districts Sululta and Wolmera districts were randomly selected from the six rural districts in the study area. In the second stage, from the lists of Peasant associations in each district, three representative peasant associations (PAs), a total of six peasant associations were randomly selected. In the third and final stage, from list of adopters and non-adopters obtained from the six selected PAs a total of 384 smallholder farm households were randomly selected using probability proportional to size sampling (PPSS) techniques.

The major tool for collecting primary data for the study was face to face interview using structured questionnaire. The questionnaire was implemented after being translated from English into “Afan Oromo,” the local language of the study area. The data was collected by trained enumerators between January 20, 2023 and April 30, 2023 after the research proposals was approved and certified by the research ethics review committee of Wollega University.

Supplementary data was found through discussion with focal groups and interview with key informants. Secondary data was gathered from published sources.

### 2.2. Methods of data analysis

In this study, the data were analyzed using both descriptive statistics and econometric models. STATA is the statistical software program utilized to analyze the data.

#### 2.2.1. Descriptive statistics.

Descriptive statistics was employed to explain the different socio-economic, institutional and other characteristics of the sample households. The statistical significance of mean differences between adopters and non-adopters smallholder farm households was tested for both continuous and dummy variables using t-tests and chi-square, respectively.

#### 2.2.2. Econometric models.

The PSM technique was employed to examine the impact of adoption of bio-fertilizer technology on faba bean productivity (yield) of the sample farm households. An accurate assessment of the influence of biofertilizer technology adoption on faba bean productivity necessitates the identification of the “average treatment effect on the treated (ATT).” This is defined as the disparity in productivity between faba bean crops cultivated by adopters and the hypothetical productivity of those crops had the adopters not adopted the technology. In this scenario, if Y denotes the productivity of faba bean and D_i_ is a dummy variable equals 1 for individuals who have adopted and 0 for those who have not, the ATT can be expressed as follows:


ATT = E[Y (1)/D=1– E[Y (0)/D=1)] (2.1)

Where: ATT reflects the impact of biofertilizer technology on the average productivity of faba bean growers who have adopted it. Y (1)/D1 indicates the average faba bean productivity among those who have adopted the technology, while Y (0)/D1 represents the average productivity of these adopters if they had not adopted biofertilizer, a value that is not observable in cross-sectional data.

Thus, since the counterfactual scenario—specifically, the productivity of their crop had they not adopted the technology—presented in the second term of equation (2.1) (E[Y (0)/ D = 1]) remains unobserved, it is essential to select an appropriate substitute to accurately estimate the ATT. Relying on the average productivity of non-adopters, which is frequently encountered in various instances, does not adequately address the issue, as there exists likelihood that the factors influencing the decision to adopt biofertilizer also impact the productivity of faba bean. Consequently, the productivity levels of both adopters and non-adopters may vary, resulting in a selection bias. To elucidate this concept, it is essential to incorporate the mean productivity of non-adopter households into equation (2.1), from which the subsequent expression can be readily obtained.


ATT=E[Y(1)/D=1]−E[Y(0)/D=1]−E[Y(0)/D=1]−E[Y(0)/D=0] (2.2)

In the previously mentioned equation (2.2), the term E[Y (0)/ D = 1]− E[Y(0)/ D = 0] signifies the presence of selection bias, which will be null if the assignment to the program was conducted randomly. This indicates that there would be no differences between the treated and control groups prior to the implementation of the program. But this is not the case in this study. Farmers of the study area decide by themselves to use or not to use (system of self selection) based on the deferent determinants of adoption decision. It is essential to establish identifying assumptions in order to assess the influence of biofertilizer on the productivity of faba beans within farming households. This study employs the PSM method to estimate the effects of biofertilizer technology on the productivity of faba beans among farmers, selecting this non-experimental approach due to the specific nature of the available data. Traditional linear regression methods may encounter issues related to multicollinearity when there is a correlation between covariates and the treatment of interest; however, the PSM technique remains unaffected by such concerns. The methodology is predicated on two key assumptions that underpin the application of PSM. The first of these is the Conditional Independence Assumption (CIA).CIA is based on the intuitive idea that knowing some variables (the conditioning set) allows one to ignore others when forecasting a target variable. In simpler terms, if anything that directly affects a result is already known, it doesn’t need additional information from other, less direct influences to comprehend that outcome [9]. In the context of impact study, CIA posits that, when controlling for a set of observable covariates that are not influenced by the treatment, the potential outcomes for the treated group (Y_1_) and the comparison group (Y_0_) are independent of the treatment assignment (2.3).


Y (0), Y (1) ⊥D/ X (2.3)

Where: ⊥ denotes independence, D/ X treatment assignments or factors affecting adoption decision of farm households. As study by Caliendo and Kopeing [10] indicates, If X does not influence the outcomes variable in this case the productivity of faba bean, then a propensity score derived from X will exhibit the same characteristic. The second assumption, referred to as the common support region, guarantees that individuals or groups sharing identical values for characteristic X possess a positive likelihood of being classified as both adopters and non-adopters of biofertilizer technology [11].The condition of a common support region facilitates the comparison of comparable units. Therefore, if the assumption of conditional independence is valid, the PSM estimator for the ATT conditional on the propensity score can be written as:


ATT=E[Y(1)/D=1,P(X)]−E[Y(0)/D=0,P(X)] (2.4)

Where:

- *Y*(1) is the mean productivity of faba bean for the adopters;
- *Y*(0) is the mean productivity of faba bean for the non-adopters (comparison group);
- *D = 1* represents using biofertilizer technology; and
- *D = 0* represents non- using of biofertilizer technology

Based on the estimated propensity score of adoption decision, among the different approaches of implementing PSM, the nearest neighbor matching (NNM) with 5-neighbors and Caliper matching are implemented in this study. To assess mean differences, a two-sample t-test is employed to evaluate whether there are significant disparities in the means of covariates between the user group and the comparison group, both prior to and after the matching process. Generally, it is expected that no significant differences in means will be observed after the matching has been completed [12].

### 2.3. Variables of the impact study

In evaluating the impact of technology adoption using PSM technique, the propensity scores is estimated using logit model. In this model the dependent variable is the probability (P_i_) of farm household i to adopt the technology. It is dummy variable equals to 1 if farm household ‘i’ is adopter and 0 otherwise.

The independent variables are those variables hypothesized to be associated with both adoption decisions and outcome variables. In this model 12 variables were hypothesized to affect both the adoption of biofertilizer technology and the outcome variables (the productivity of faba bean crops) which include: Educational level of the house hold head (EDLHH), Gender of the head (GNDR), Age of the head (AGE), Number of oxen owned (OXEN), Access to information (INFN), Size of farm in hectare (FRMS), Access to Credit (CRDT), Perception of household head on the technology (PERC), Income of the household (INCM), Availability of labor (ALBR), Extension contact (EXTNC), Involvement of the house hold head in the off-farm activity (OFFRM) and Training (TRNG).

In the outcome model the dependent variable of the impact evaluation of the adoption of biofertilizer technology on the productivity of farm households is the ATT. ATT refers to the impact of biofertilizer technology adoption on the productivity of faba bean on the plots of smallholder farmers, It represents the mean difference in faba bean productivity with and without the technology represented by E[Y1/D = 1, P(X)] and E [Y0/ D = 0, P(X)] respectively in equation 2.5.


ATT=E[Y1/D=1,P(X)]−E[Y0/D=0,P(X)] (2.5)

The independent variables are therefore the productivity of faba bean on adopters’ plots (*Y*_1_) and the productivity of faba bean on the comparison groups’ plots (*Y*_0_).

## 3. Results and discussion

The results of descriptive and econometric analysis are presented in this section. The first portion discusses the results of descriptive statistics, while the second section discusses the results of the econometrics model. STATA software was used to analyze both sets of statistics.

### 3.1. Results of descriptive statistics

The results of descriptive statistics indicates that there is a statistically significant mean differences in educational level of household head, gender of the household head, age of household head, number of oxen owned, access to information, size of farm, perception of household head on the technology, income of the household, availability of labor, number of extension contacts, involvement of the household head in the off/non-farm activities, and access to training between adopters (treated) and non adopters (untreated) of biofertilizer technology in the study area. However, it was found that there was no significant difference between the two groups’ mean values for access to credit, Table 1. As indicated in the table on average adopters are educated, mainly male headed, younger, has more oxen, larger farm size, has more access to credit and information, higher income than non-adopter farm households. Non-adopters have on average larger number of labour and participated in less number of training out of their locality.

**Table 1:** The mean differences between adopters and non adopters before matching.

Variables	Mean		Mean Diff
	Non-adopters	Adopters	
Educational level of household head (Years)	2.344	7.219	−4.875***
Gender of the household head(M = 1, F = 0)	0.828	0.938	−0.109***
Age of household head (Year)	49.1	41.3	7.766***
Number of oxen owned (Number)	1.760	2.760	−1.000***
Access to information (yes = 1, no = 0)	0.490	0.979	−0.490***
Farm Size (hectare)	2.912	3.540	−0.628***
Access to Credit(yes = 1, no = 0)	0.260	0.323	−0.063^NS^
Perception of household head on the technology (yes = 1, no = 0)	0.167	0.984	−0.818***
Income of the household (Ethiopian Birr)	300,000	360,000	−60,000***
Availability of labor (Number)	3.098	2.913	0.185*
Extension contacts (Number)	2.490	6.880	−4.391***
Involvement of the household head in the off/non-farm activities	0.255	0.656	−0.401***
Training (Number)	2.026	5.333	−3.307***

Note: *** and * indicate the level of significance 1 and 10 percent, respectively.

Source: Own computation, 2024.

But it is evident that after matching, these before-matching mean differences become insignificant. This indicates that the PSM method effectively creates, through experimental processes, a non-adopters group that is comparable, allowing for a meaningful comparison of the productivity of faba bean between the adopters of biofertilizer technology and the non-adopters group.

### 3.2. Econometric results

This section refers to the analysis of the effect of adoption of biofertilizer technology on the smallholder farmers’ productivity of faba bean crop. This study assesses the causal impact of biofertilizer technology on the productivity of faba bean crops on the plots of smallholder farmers, utilizing the PSM procedure. The analysis incorporates Nearest 5 Neighbour matching algorithms alongside caliper methods, executed through the psmatch2 command on the STATA platform. Subsequently, the findings related to the estimation of propensity scores, the ATT, and the quality analyses following the matching process are discussed.

#### 3.2.1. The assessment of the propensity score.

Propensity score is the likelihood that an individual chooses the treatment based on certain conditions. In this study, it estimates the probability of an individual farmer being an adopter or non-adopter of biofertilizer technology using a logit model based on certain observable covariates. The model took into account all measurable covariates that influence the probability of adoption of the technology and the productivity of faba beans, utilizing the available observational data. The results presented in Table 2 indicate that the logit model is statistically significant. The analysis reveals a significant statistical difference between adopters and non-adopters households, in terms of the following factors. These factors include the educational level of the household head (EDLHH), the age of the household head (AGE), the household head’s perception of the technology (PERC), the frequency of extension contact (EXTNC), the household head’s participation in off-farm or non-farm activities (OFFRM), and the number of training received (TRNG).

**Table 2:** Estimation of propensity score.

Adoption Status		Coef.	Std. Err.	z
Educational level of HH (Years)		0.14	0.048	2.84*
Gender of the HH (M = 1, F = 0)		0.29	0.731	0.40
Age of HH(Year)		−0.05	0.017	−2.91*
Number of oxen owned (Number)		−0.06	0.155	0.40
Access to information (yes = 1, no = 0)		0.61	0.556	1.09
Size of land (hectare)		−0.08	0.141	−0.56
Access to Credit(yes = 1, no = 0)		−0.37	0.335	−1.09
Perception of household head on the technology (yes = 1, no = 0)		2.06	0.401	5.14*
Income of the household (Ethiopian Birr)		1.24	1.245	0.99
Availability of labor (Number)		−0.21	0.152	−1.37
Extension contacts (Number)		0.34	.092	3.73*
The household head’s participation in off-farm or non-farm activities (yes = 1, no = 0)		1.06	.3634592	2.91*
Training (Number)		0.32	.1140597	2.77*
cons		−2.80	1.220514	−2.29**
LR chi2(13)	440.21	Log likelihood	−46.06	
Number of obs	384	Pseudo R2	0.83	
Prob > chi2	0.00			

Source: Model result from own survey data, 2024.

Note: ** and * indicate the level of significance 5 and 10 percent, respectively.

HH = Household Head.

The table illustrates that these factors contributed to the varying levels of household participation in the adoption of biofertilizer technology. The specifics regarding the influence of each covariate on the adoption decisions made by farm households are not elaborated upon, as the primary objective is to compute the propensity scores, which were utilized in the following matching process.

#### 3.2.2. Average treatment effect on the treated (ATT).

The ATT is estimated through the application of nearest neighbors matching, specifically utilizing the 5-Nearest Neighbors matching algorithms alongside caliper matching techniques. The findings are detailed in Table 3, which not only displays the mean values of the outcome variable (the productivity of faba bean) but also highlights the mean differences between the treated (adopters) and the control groups. It is important to note that the analysis in this section primarily relies on the results derived from the nearest neighbor matching algorithm, as it demonstrated a greater degree of statistical significance compared to the caliper method.

**Table 3:** Nearest neighbor and Caliper matching results of ATT.

Outcome variable is Log of the productivity of faba bean (LPRDTY)						
Matching procedure	Sample	Treated	Control	Differences	S.E	T-Test
Nearest 5 neighbor	ATT	3.152	2.448	0.704	0.163	4.33***
Caliper	ATT	3.047	2.632	0.415	0.180	2.31**

Source: Model results, 2024.

*** and ** Significant at 1% and 5% level of significance respectively.

The findings indicate a statistically significant enhancement in the productivity of faba bean attributable to the adoption of biofertilizer technology among smallholder faba bean producers in the districts surrounding the capital city of Ethiopia, Addis Ababa. Specifically, it was observed that households using biofertilizer technology on their plots achieved an average annual yield that is 5.1 quintals per hectare higher than that of households not utilizing this technology, as derived from the antilog of 0.704. This notable outcome suggests that the adoption of biofertilizer technology significantly boosts the productivity of faba bean among farmers in the study region. These results align with prior empirical studies referenced in sources [13–16].

#### 3.2.3. Assessment of the quality of matching.

The effectiveness of matching estimations is fundamentally influenced by the validity of the PSM assumption regarding conditional independence, which guarantees that the treatment or adopters and control groups are as comparable as possible. In this study, the matching quality analyses were conducted utilizing the t-test alongside mean or median bias. The primary assessment for evaluating the quality of the matching results involved comparing the samples before and after the matching process to determine whether significant differences still exist between adopters and non-adopters. Table 4 presents the mean test results for smallholder farm households categorized as treated (adopters) and those that were not treated (non-adopters), based solely on matched observations. While it is anticipated that differences should be present prior to the matching process, it is essential that, after matching, the variables achieve balance within both the adopter and non-adopter groups, thereby ensuring that no significant disparities are identified [12].

**Table 4:** T-test for the mean differences of matched sample.

Variable	Mean		Differences	t-test	
	Treated	Control		t-stat	P > |t|
Educational level of household head (EDLHH)	4.567	5.413	−0.846	−0.93	0.356
Gender of the HH (GNDR)	0.967	0.973	−0.006	−0.15	0.882
Age of household head (AGE)	47.233	45.047	2.186	0.98	0.332
Number of oxen owned (OXEN)	2.300	2.353	−0.053	−0.19	0.848
Access to information (INFN)	0.967	0.907	0.06	0.95	0.348
Farm Size in hectare (FRMS)	3.000	3.000	0	−0.29	0.777
Access to Credit (CRDT)	0.467	0.38	0.087	0.67	0.505
Perception of household head on the technology (PERC)	0.900	0.807	0.093	1.01	0.315
Income of the household (INCM)	340,000	340,000	0	0.03	0.979
Availability of labor (ALBR)	3.090	3.037	0.053	0.18	0.860
Extension contact (EXTNC)	3.233	3.173	0.06	0.17	0.867
Training (TRNG)	2.967	2.9067	0.0603	0.16	0.877

Source: own computation, 2024.

As a result, as Table 1 indicates, there were significant mean differences between the adopters and non-adopters groups before matching in all covariates except access to credit. However, Table 4 indicates that none of these covariates has a significant mean difference between the two groups after matching. This clearly shows the high quality of the matching property for the smallholder farmer’s matched samples of faba bean productivity.

Sianesi [16] proposes an alternative method for evaluating the effectiveness of the matching procedure. The author recommends a comparison of the pseudo-R^2^ obtained from the matched sample with that derived from a model utilizing all observations, followed by a re-estimation of the logit model based exclusively on the matched observations. It is expected that, post-matching, there will be no substantial differences in the covariate distributions between the treated and non-treated groups. Consequently, the pseudo-R^2^ for the matched sample should not exhibit a significant deviation from zero. Table 5 illustrates the variation in this statistic, affirming that the matched sample shows no significant differences in covariate distributions between those who adopted and those who did not adopt biofertilizer technology. Furthermore, the table indicates a notable reduction in both the mean and median bias following the matching process, suggesting that the covariate distributions within the matched sample were effectively balanced through the matching procedure.

**Table 5:** Pseudo-R^2^ before and after matching.

Sample	Pseudo -R^2^	LR chi2	p > chi2	MeanBias	MedBias
Unmatched	0.809	430.77	0.000	107.7	89.2
Matched	0.038	3.18	0.994	12.2	5.9

Source: Own computation, 2024.

## 4. Conclusions and policy implications

The productivity of faba beans produced by smallholder farmers in the central highlands of Ethiopia is examined in this study as a direct impact of the use of biofertilizer technology. The data used in the investigation was collected from 384 small-holder farmers in six rural districts surrounding Addis Ababa; the capital city of Ethiopia. The results indicate that smallholders in the research areas had higher levels of faba bean productivity as a result of the adoption of biofertilizer technology. According to the PSM result, biofertilizer adoption on average increased faba bean productivity of adopters by about 5.1 quintals ha^-1^ than the non-adopters.

This implies that it is preferable to enhance the biofertilizer technology utility in order to boost faba bean productivity on smallholder farm households’ plots. Furthermore, it is recommendable to reinforce biofertilizer technology promotion, extension, and adoption actions.

## Supporting information

S1 File Impact study data. (XLSX)

S2 File Explanation of biofertilizer technology. (DOCX)
