# Supplementary material for: The impact of biofertilizer technology adoption on the faba bean productivity in the central highlands of Ethiopia: The propensity score matching model
Source: PLoS One. 2025 Sep 29;20(9):e0333105. doi: 10.1371/journal.pone.0333105 (PMC12478907; doi:10.1371/journal.pone.0333105)
Supplement: S2 File — (DOCX) [file pone.0333105.s002.docx]

# S2. Explanation of biofertilizer technology

Contents

[S2. Explanation of biofertilizer technology 1](#_Toc209001934)

[1. Introduction 1](#_Toc209001935)

[2. The concept of bio-fertilizers 1](#_Toc209001936)

[3. The type and benefits of bio-fertilizer technology used in our study 1](#_Toc209001937)

[4. Supply of rhizobial bio-fertilizers in Ethiopia 3](#_Toc209001938)

[5. Distribution of bio-fertilizer in the study area 4](#_Toc209001939)

[6. Summary 5](#_Toc209001940)

[7. References 6](#_Toc209001941)

# Introduction

In response to the final remarks made by "reviewer #1," we are writing this brief history of the type of biofertilizer that was used in our study. This writing is organized into five sections. The first section presents the introduction. The definition and key roles of biofertilizer in agriculture are described in the second section, which also highlights the overall idea of biofertilizer. The ***biofertilizer technology used in our study***, specifically the type adopted by faba bean growers in the study area, is discussed in the third section. Section four explains the supply of ***rhizobial*** biofertilizer technology and presents the major companies producing and distributing it. Distributions of biofertilizers among the districts of the study area is identified in the fifth section. Finally, a summary is presented.

# The concept of bio-fertilizers

Bio-fertilizers are natural fertilizers that are living microbial inoculants of bacteria, algae, and fungi alone or in combination, and they augment the availability of nutrients to the plants, which may help in increasing crop productivity ([Vishal and Abhishek, 2014](#Vishal)).

Biofertilizers play unique roles in agriculture, especially given the current high cost of chemical fertilizers and their hazardous effects on soil and human health ([Kumar *et al*., 2017](#Kumar)). Biofertilizers enhance plant growth when introduced to the soil, seeds, or plant surfaces, as they consist of beneficial live bacteria combined with cost-effective carrier materials. These biologically active formulations enhance the availability of essential nutrients such as nitrogen (N), phosphorus (P), and potassium (K), which are crucial for promoting plant growth ([Suyal, 2016](#Suyal)).

Biofertilizers show plant growth-promoting qualities and increase the yield by various mechanisms like nitrogen fixation, K-solubilization, P-solubilization and mobilization, micronutrient solubilization, plant growth promotion, preventing the depletion of the soil organic matter, and maintenance of the natural habitat of the soil ([Jeyabal and Kupuswamy, 2001](#Jayabal)). Additionally, it is well known to be eco-friendly and environmentally safe ([Siczek, 2016](#Siczek)).

# The type and benefits of bio-fertilizer technology used in our study

According to a research report by [Jabasingh (2018)](#Jabasingh), three main types of biofertilizer strains identified for several East African countries, including Ethiopia, are Rhizobia (Rhizobium leguminosarum L.), Pseudomonas (Pseudomonas putida), and Mycorrhiza (Glomus sp.).

Rhizobial biofertilizer enhances nutrient flow to pulse crops and is considered one of the most effective and sustainable methods for managing soil fertility in the Ethiopian highlands. Rhizobia are bacteria that assist in nitrogen fixation by establishing themselves inside the root nodules of legumes. This process helps replenish soil nutrients and allows them to function as biofertilizers. Nowadays, many rhizobium strains are being produced and supplied for faba bean, chickpea, lentil, field pea, common bean, and soybean production ([Fassil, *et al*., 2018](#Fassil)).

The application of rhizobial biofertilizers has great economic importance in improving the productivity of pulse crops of smallholder farmers in Ethiopia ([Shita *et al*., 2018](#Shita)). Research report by [Jabasingh (2018)](https://quillbot.com/grammar-check/d/031ab439-f4ad-4218-9e9a-088eff3f5a30#Jabasingh) indicated that generally in Ethiopia, on average it is possible to increase the yield of pulse crops by at least 30% on farmer’s field if bio-fertilizer is used. Our study identified the impact of rhizobial biofertilizer technology adoption on the productivity of faba bean in the central highlands of Ethiopia. Thus the type of biofertilizer technology used in our study entitled *“The impact of biofertilizer technology adoption on the faba bean productivity in the central highlands of Ethiopia”* is ***rhizobial*** biofertilizers. As indicated in the manuscript the finding of the study revealed that, **rhizobial** biofertilizer adoption on average increased faba bean productivity of adopters by about 5.1 quintals ha^-1^ than the non-adopters smallholder farm households in the central highlands of Ethiopia.

The agricultural and environmental benefits of rhizobial biofertilizers in Ethiopia were summed up by [Getahun *et al.* (2020)](#Getahun) as follows: soil health and fertility improvement, yield improvement, economic benefit, and environmental benefit.

1. **Improvement of soil health and fertility**: if the roots of the legumes are left in the ground, the ongoing application of rhizobial biofertilizers in cropping systems helps to improve soil fertility status by raising soil nitrogen levels and soil health (by increasing the growth of other beneficial soil microorganisms such as fungi, actinomycetes, bacteria, etc.). It also enhances soil organic matter as root and leaf drop are systematically worked into the soil.
2. **Yield improvement**: Rhizobial biofertilizers contribute to increased grain legume crop productivity and yield. In any cropping system, it increases grain or biomass yield by up to 10% (especially with 100 kg Di Ammonium Phosphate ha-1) by promoting plant growth and enzymes, hormones, and auxins. When favorable markets for the farm's products are present, higher yields translate into higher profits. Additionally, it enhances grain legume crops' protein quality. Furthermore, the residual effect of rhizobial biofertilizers increases soil nitrogen and nutrient availability due to better stover and straw yield, which ultimately increases yield on Striga control.
3. **Economic benefit:** Farmers frequently experience direct and indirect cost reductions even if there is a little labor cost associated with inoculating the seeds before to planting. By adding rhizobial biofertilizers to inorganic nitrogen fertilizer, the cost of producing legume crops is decreased. For instance, using 500 g of rhizobial biofertilizer—enough to coat the seed needed to plant a hectare of land—is ten times less expensive than using 50 kg of urea for the same purpose. Compared to inorganic fertilizers, it is less expensive and easier to transport and store due to its small pack sizes (sachets). Since it is an authorized input for organic farming, products may fetch higher prices, particularly for exportable bean crops.
4. **Environmental benefit**: this technology reduces soil and water pollution compared to chemical fertilizers from manufacturing to its use.

In Ethiopia, much effort has been devoted to identify and characterize the efficient forms of local and exotic Rhizobial strains for pulse crops ([Jabasingh, 2018](#Jabasingh)). After intensive screening under laboratory, greenhouse, and field conditions, elite strains have been put under mass production and supplied to farmers in various parts of the country since 2006 ([Asfaw and Angaw, 2006](#Asfaw)).

Research on biofertilizer in Ethiopia has primarily concentrated on rhizobium and related genera, and it is divided into three distinct phases.

In ***the first and initial phase (1980-1990)***, Rhizobium research was initiated by the National Soil Research Center and the Holeta Agricultural Research Center, which were then part of the Institute of Agricultural Research. Their primary focus was on the collection, isolation, and study of the symbiotic effectiveness of faba bean ([Desta and Angaw, 1986](#Desta); [Asfaw and Angaw, 2006](#Asfaw)).

In the **second and middle phase** (1991-2005), universities such as Hawassa and Addis Ababa have started intensified research on local strains of major legumes such as faba bean, chickpea, soybean, haricot bean, and lentil. Field demonstration works have been conducted at Jimma and Kulumsa research centers ([Desta et al., 2006](#Desta2)).

In the **third and recent phase** (2006-present), further research on the genetic diversity and phylogenetic relationships of rhizobia isolated from root nodules continued. Also in this phase mainly mass production and distribution / commercialization of bio-fertilizer were launched by several private companies ([Endalkachew et al., 2009](#Endalkachew) and [Nigusie](#Nigusie), 2020).

# Supply of rhizobial bio-fertilizers in Ethiopia

Several companies in Ethiopia are involved in the production and distribution of rhizobial bio-fertilizers. Menagesha Biotech Industry (MBI) and Bio-Safe Bio Fertilizer Manufacturing (BBM) are the two major private companies engaged in the production and distribution of bio-fertilizers, focusing on rhizobium inoculants for pulse crops. Additionally, the National Soils Testing Center (NSTC) and some agricultural research centers are also involved in these biofertilizer production and distribution. Furthermore, the government has been supporting the establishment of additional biofertilizer production units in the country.

Menagesha Biotech Industry (MBI) is a private bio-fertilizer producing company established in 2012 in Ethiopia with the objective of producing and distributing bio-fertilizers and long run vision of becoming the leading bio-fertilizer producing industry in the region. The production capacity of the company was 102,000 (125 grams each enough for quarter hectare) of inoculants. As a penetration strategy to the inoculants business, the company has been supplying inoculants for a minimum steady price and also in the form of credit where defaulting was a key challenge. The Ministry of Agriculture at federal level and Bureaus of Agriculture at regional, zonal and district levels were approached to be a bridge between the company and smallholder famers ([Fassil et al., 2018](#Fassil)).

Bio-Safe Bio Fertilizer Manufacturing (BBM) is the second major private company established in 2019; it is one of the major producers of biofertilizer (rhizobium inoculants) in Ethiopia. They produce and distribute bio-fertilizers for major legume crops like faba bean, field pea, chickpea, lentil, haricot bean, and soybean. With the ability to produce more as the market expands, BBM has a substantial manufacturing capacity of approximately 100,000 packets per year, which is sufficient to inoculate 25,000 hectares (1 packet (125 g) covers 0.25 hectares). In the three main regions of the country (Amhara, Oromia, and Southern regions), BBM sold 353,689 sachets of rhizobium inoculants for a variety of pulse crops between 2021 and 2024. This covers approximately 88,422.25 hectares of land used for pulse crops such as faba beans. Currently, the company has the plan to expand its business to produce more inoculants for legume crops and non-legume crops such as barley, wheat, maize, and teff. BBM intends to collaborate closely with research institutions and other partners to conduct sample work for producing new inoculants for non-legume crops. For this new initiative, the company will perform cost estimation and consider equity financing, as well as partner others to conduct the research work and laboratory testing. (<https://casaprogramme.com/bio-safe-bio-fertilizer-manufacturing>,-(BBM))

Other emerging minor biofertilizer-producing companies in Ethiopia are Green Ethiopia, Eco-green, Re-nature, etc. Green Ethiopia, an organic fertilizer producer, was founded in 2022 and is based in Addis Ababa, Ethiopia. This company produces organic fertilizers using advanced composting technologies and local agricultural and urban waste. Their products are designed to be high nitrogen, phosphorus, and potassium (NPK) and water-retaining, reducing irrigation needs.

## Distribution of bio-fertilizer in the study area

Biofertilizer, broad bed maker, compost, and others are among the major new agricultural technologies used in the six rural districts in the study area during the 2021/2022 cropping season. The zonal and district agricultural offices have the responsibility to plan, supply, and control the distribution and application of these technologies. The smallholder farmers of the study area have used rhizobial biofertilizer technology to produce faba beans. A total of 2,621 sachets of biofertilizer were used by smallholder faba bean growers in the six districts during the 2021–2022 cropping season, according to the annual reports from each district in the study area. The districts of Wolmera, Sululta, Mulo, Barack, Sebata Hawas, and Akaki used 898 sachets (34%), 570 sachets (22%), 450 sachets (17%), 320 sachets (12%), 307 sachets (12%), and 76 sachets (3%) respectively. As illustrated in Figure 9, Wolmera and Sululta districts were the highest users of the technology in the area, accounting for 56% of all the biofertilizer technology employed in this crop season.

Figure 1: Total bio-fertilizers distributed in the study area in 2021/22 crop season

Source: Own computation (2022)

# Summary

Bio-fertilizers are natural fertilizers that enhance the availability of nutrients to the plants, which help in increasing the productivity of crops. Compared to chemical fertilizers, bio-fertilizers are more economical and accessible for smallholder farmers, more environmentally friendly, and easier to transport. Research on biofertilizers in Ethiopia has primarily focused on rhizobium and related genera. This research began in the 1980s, with a specific emphasis on rhizobial biofertilizers. Rhizobial biofertilizer is one of the most effective and sustainable methods for managing soil fertility in the Ethiopian highlands. It is particularly beneficial for increasing the productivity of pulse crops, including faba beans, field peas, soybeans, and others. Several commercial companies are currently involved in the production and distribution of biofertilizer in Ethiopia. The major objective of our study was to analyze the impact of rhizobial biofertilizer on faba bean productivity in the central highlands of Ethiopia.

# References

Asfaw Hailemariam and Angaw Tsigie, (2006).Biological Nitrogen Fixation Research in Food Legumes in Ethiopia.EIAR and ICARDA, Addis Ababa, Ethiopia.

Desta Beyene and Angaw Tsigie, (1986). Exhaustion trial on cereals, pulses and oil crops in Holeta Progress Report, pp. 226-228, IAR, Addis Ababa, Ethiopia.

Desta Beyene, Serawit Kassa, Ampy, F., AmhaAsefa and Tadesse Gebremedhin (2006). Ethiopian soils harbor natural populations of rhizobia that form symbioses with common bean (Phaseolus vulgaris L.). Arch. Microbiol. 181: 129-136, IAR, Addis Ababa, Ethiopia.

Endalkachew Wolde-meskel, Zewdu Terefework, Lindström, K. and Frostegård, Å. (2009). Genetic diversity and phylogeny of rhizobia isolated from agro-forestry legume species in southern Ethiopia. Int. J. Syst. Evol. Microbiol. 55:1439-1452. IAR, Addis Ababa, Ethiopia.

Fassil Assefa, Gemechu Keneni, and Negash Demissie (2018). Overview of Rhizobial Inoculants Research and Biofertilizer Production for Increased Yield of Food Legumes in Ethiopia. *Ethiop. J. Crop Sci. Special Issue Vol. 6 No.3 (2018).*

Getahun Mitiku , Abere Mnalku, James Watiti (2020). Application Guideline for Rhizobial Biofertilizer Technologies. Ethiopian Institute of Agricultural Research Application Guideline for Rhizobial Biofertililizer. February 2020. DOI: 10.13140/RG.2.2.15484.90249.

Jabasingh, S. Anuradha (2018). Fertilizer application status in Ethiopia, with a special emphasis on biofertilizer. Publisher:*DOI:* [*https://doi.org/10.31579/2578-8825/2018*](https://doi.org/10.31579/2578-8825/2018).

Jayabal, A. and Kuppuswamy, G. (2001). Recycling of organic wastes for the production of vermicompost and its response in rice–legume cropping system and soil fertility. *European Journal of Agronomy, Volume 15, Issue 13. DOI:*[10.1016/S1161-0301(00)00100-3](https://ui.adsabs.harvard.edu/link_gateway/2001EuJAg..15..153J/doi:10.1016/S1161-0301(00)00100-3).

Kumar, R., Narendra Kumawat and Yogesh Kumar Sahu (2017): Role of Bio-fertilizers in Agriculture. Popular Kheti, Volume -5, Issue-4 (October-December), 2017 India. Available on line at www.popularkheti.info.

Nigusie Alemayehu (2020). The use of biofertilizer by smallholder farmers and its impact on productivity of pulse-cerial cropping system in Arsizone, oromia regional state, Southeastern Ethiopia. East African Journal of Sciences, 14(1);1-12, Addis Ababa, Ethiopia.

Shita, A., Nand Kumar & Seema Singh(2018). Agricultural Technology Adoption and its Determinants in Ethiopia: 2018, A Reviewed Paper. Vol: I. Issue LVV; [www.apjor.com](http://www.apjor.com) .

Siczek, A., Lipiec, J. (2016). Impact of faba bean-seed rhizobial inoculation on microbial activity in the rhizosphere soil during growing season. International Journal of Molecular Sciences, 17: 784.

Suyal, D.C., Soni, R., Sai, S., Goel, R. (2016). Microbial Inoculants as Biofertilizer. In: Singh, D., Singh, H., Prabha, R. (eds) Microbial Inoculants in Sustainable Agricultural Productivity. Springer, New Delhi. https://doi.org/10.1007/978-81-322-2647-5_18 J.K. Vessey, Plant growth promoting rhizobacteria as biofertilizers, Plant Soil 255 (2003) 571–586, doi: 10.1023/A:1026037216893.

Vishal, K. D., & Abhishek, C. (2014). Isolation and characterization of Rhizobium leguminosarum from root nodules of Pisums sativum L. Journal of Academic and Industrial Research, 2(8), 464-467. Vol 2. Rhizobium, pp 1-34, (Broughton, W.J., ed). Oxford University Press, UK.
